# Supplementary material for: Evidence supported by Mendelian randomization: impact on inflammatory factors in knee osteoarthritis
Source: Front Med (Lausanne). 2024 May 28;11:1382836. doi: 10.3389/fmed.2024.1382836 (PMC11165061; doi:10.3389/fmed.2024.1382836)
Supplement: Supplementary file 1 [file Data_Sheet_1.ZIP › data/GCST90274821_5e6/821.leaveoneout.pdf]

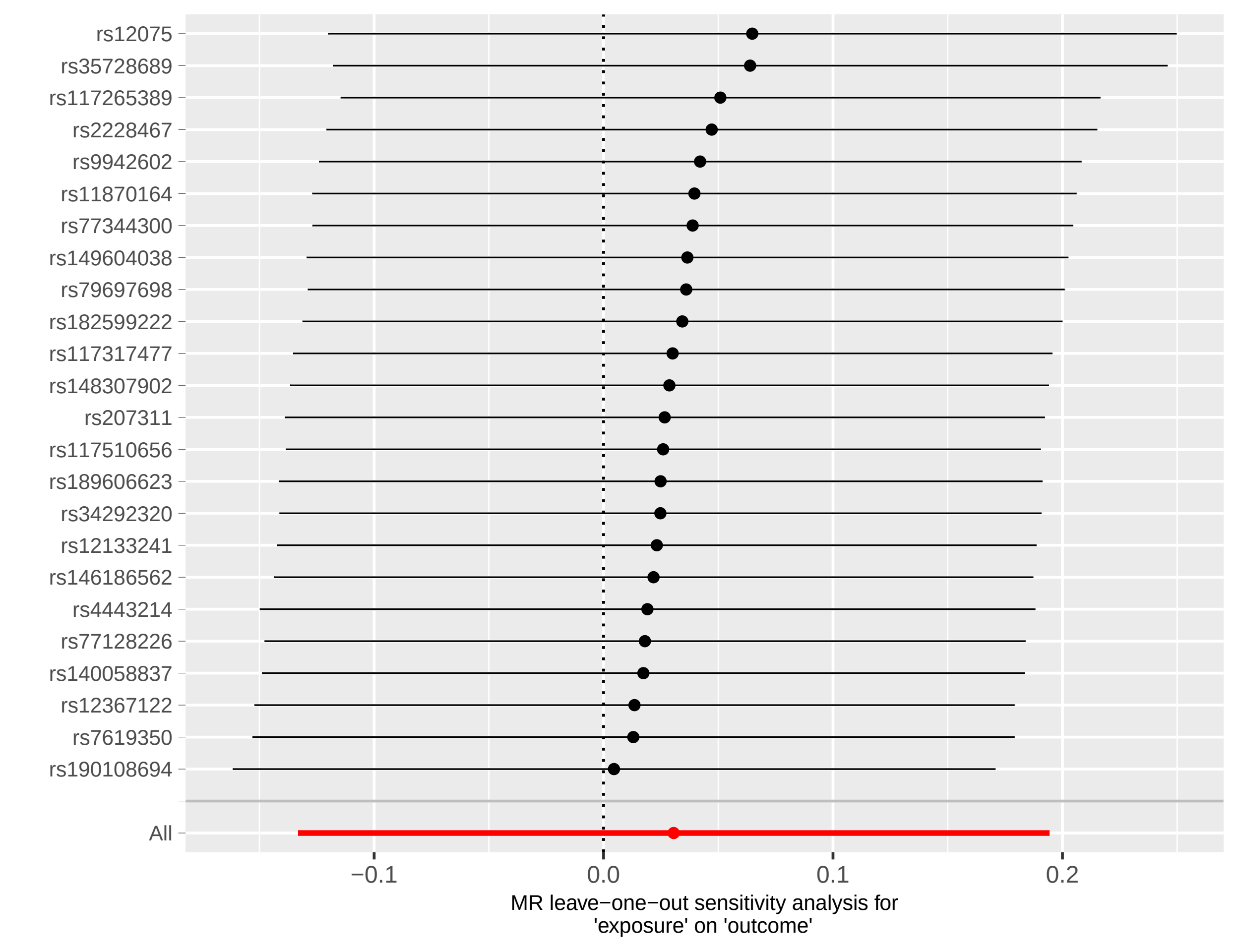
rs12075
rs35728689
rs117265389
rs2228467
rs9942602
rs11870164
rs77344300
rs149604038
rs79697698
rs182599222
rs117317477
rs148307902
rs207311
rs117510656
rs189606623
rs34292320
rs12133241
rs146186562
rs4443214
rs77128226
rs140058837
rs12367122
rs7619350
rs190108694
All
−0.1
0.0
0.1
0.2
MR leave−one−out sensitivity analysis for
'exposure' on 'outcome'
